# Analyzing video recorded support of postnatal transition in preterm infants following a c-section

**DOI:** 10.1186/s12884-016-1045-2

**Published:** 2016-08-25

**Authors:** Dimitrios Konstantelos, Jürgen Dinger, Sascha Ifflaender, Mario Rüdiger

**Affiliations:** Department of Neonatology and Pediatric Intensive Care, Medizinische Fakultät Carl Gustav Carus, TU Dresden, Fetscherstraße 74, Dresden, 01307 Germany

**Keywords:** Delivery room, Video recording, Management, Preterm

## Abstract

**Background:**

Over the past years, research on neonatal resuscitation has focused on single interventions. The present study was performed to analyze the process quality of delivery room management of preterm infants born by c-section in our institution.

**Methods:**

We performed a cross-sectional study of videos of preterm infants born by c-section. Videos were analyzed according to time point, duration and number of performed medical interventions. The study period occurred between January 2012 and December 2013. Infants were caterogized in 3 groups according to their gestational age.

**Results:**

One hundred eleven videos were analyzed. 100 (90 %) of the infants were transferred to NICU and 91 (83 %) received respiratory support after a median of 0.5 min. All infants were auscultated after 8 (5–16) seconds median (IQR) and an oxygen saturation sensor was placed after 37 (28–52) seconds. 23 infants were intubated after 9 (6–17) minutes and 17 received exogenous surfactant; 29 % according to INSURE (intubation-surfactant-extubation) technique. The duration of intubation attempts was 47 (25–60) seconds. 51 % of the newborns received a sustained inflation for 8 (6–9) seconds. A successful IV-line placement occurred after 15 (12–20) minutes. 4 % of the infants were transported to the NICU without an IV-line after 3 (difference range: 2–5) unsuccessful attempts.

**Conclusions:**

Using video analysis as a tool to study process quality, we conclude that interventions differ not only between but also within similar age groups. This data can be used for benchmarking with current guidelines and practice in other centers.

**Electronic supplementary material:**

The online version of this article (doi:10.1186/s12884-016-1045-2) contains supplementary material, which is available to authorized users.

## Background

Postnatal adaptation during the first “Golden Hour of Life” represents a crucial time. Especially on immature newborns on whom quality of delivery room (DR) management is of great importance.

Thus, research has focused on optimizing postnatal adaptation [1–3]. New concepts of respiratory support during the DR-management such as continuous positive airway pressure (CPAP) [4–6], sustained inflations (SI) [7–11], and less invasive surfactant administration have been studied [12–14]. Furthermore, the importance of monitoring – not only of vital signs but also respiratory parameters or body temperature – has been proven in clinical studies [15, 16]. Finally, interventions that have been widely used in the past – such as suctioning of the oropharynx – have been now abandoned [17].

However, there is not a single “magic bullet” to improve DR-management of preterm infants [18, 19]. In order to reach an improvement outcome, the entire approach has to be changed from an aggressive resuscitation towards a gentle support of transition, which requires multimodal management [20, 21].

In an attempt to improve the outcome of preterm infants in our institution, we implemented a quality assurance program for DR-management [22–24]. The current study was performed to identify differences in current DR-management of preterms within and between different gestational age (GA) groups.

## Methods

### DR-management

In our institution, postnatal transition of a preterm infant is supported by a caregiving team consisting of a neonatologist, a pediatric resident (or neonatal fellow) and a NICU nurse. After birth the midwife places the infant onto a scale to be weighed, thereafter the NICU-nurse places the infant under a radiant heater. Newborns weighing less than 1500 g are placed in a plastic wrap. The newborn management room has a temperature of 24–26° Celsius.

DR-management should adhere to local guidelines, which are in accordance with international recommendations. In short, the neonatologist in charge is standing behind the head of the newborn and is responsible for the respiratory support. The second caregiver – responsible for initial auscultation – is standing on the left side of the newborn and shows the newborn’s heartbeat frequency by moving his finger until both a reliable heart rate and saturation readings are available. If the newborn is in need of a respiratory support, CPAP is administered via a facemask with a pressure of 6–8 cm H_2_0. In case of an initial bradycardia (<100 bpm) a sustained inflation with a pressure of 20 cm H_2_0 for 10 s is applied up to a maximum of 3 times. If bradycardia persists, positive pressure ventilation via a facemask is commenced. After respiratory stabilization of the newborn an intubation-surfactant-extubation (INSURE) procedure is performed if the predefined criteria are met. Prior to transferring the infant to the NICU, an intravenous line (IV-line) is placed, blood is collected, glucose infusion is administered and the newborn is shown to their mother.

### Video recording

Video recording of DR-management was performed as previously described [22–24]. For the present study, all recordings of preterm infants (<37 GA) born after c-section in 2012 and 2013 during the morning shift on working days were analyzed. Videos of infants with known congenital malformation were excluded. Analysis started with the arrival of the newborn under the radiant heater (Time point 0), which takes place usually about 20 s after cord clamping [22].

For the current analysis we distinguished the infants according to their gestational age arbitrarily in the following three groups: low gestational age (LGA): ≥ 34 GA, very low gestational age (VLGA): 31^+0^ – 33^+6^, extremely low gestational age (ELGA): <31 GA. For infants that stayed in the delivery room, analysis ended either at transfer to the mother or after 10 min (whichever of the two preceded the other). For infants in need of a further treatment in NICU, analysis was extended until transfer to the NICU.

### Analysis of data

The following medical interventions were analyzed with regard to start time, total duration and frequency of occurrence.

#### Routine care

All routine interventions were performed either to assess or stabilize the infant’s condition (auscultation, finger heart rate sign, new towel or plastic wrap placement to prevent heat loss, placement of saturation sensor, temperature management).

#### Respiratory support

CPAP administration via a facemask, endotracheal or nasopharyngeal tube (which is mainly used during NICU transfer), SI, ventilation with consecutive inflations via a facemask or through an endotracheal tube, intubation (defined as the time that laryngoscope stayed in infants mouth), surfactant administration, suctioning.

#### IV-line placement

Time point and duration (defined as the time between end of disinfection of the skin and stabilization of the IV-line with a band) of successful and unsuccessful IV-line placement attempts were determined.

#### Other interventions

The total time during which the infant was not manipulated was calculated, were manipulations are defined as any of the above-described interventions or any other contact with the infant’s skin.

### Statistical analysis

Descriptive data are presented as median (interquartile range, IQR). Interventions were analyzed with respect to parameters such as time of first occurrence, duration and frequency. Two box-plot figures were used to display the variance with respect to the time point of first occurrence.

### STROBE statement

We confirm that our research complies with the STROBE [Strengthening the Reporting of Observational Studies in Epidemiology (collaboration)] guidelines.

## Results

One hundred eleven videos of DR-management in preterm infants born by c-section were analyzed. We excluded 5 videos due to congenital malformations. Videos had a total duration of 2619 min [median of 24 (IQR, 19–29) minutes per video]. 22 different caregivers performed DR-management on these infants with a median frequency of 2 (IQR 1–5) patients per caregiver. 50 videos of LGA, 46 of VLGA and 15 of ELGA infants were analyzed. Only 11 of the 111 (9.9 %) preterm infants could remain in the delivery room with their mother. These infants had a median gestational age of 35^+6^ weeks (IQR, 35^+5^ – 36^+5^). The remaining 100 infants [GA 33^+1^ (32 ^–^ 34^+4^)] were transferred for further treatment after a median of 24 (19–29) minutes (Table 1).

**Table 1:** Median (IQR) duration of various events

	LGA		VLGA		ELGA	
	n		n		n	
Timepoint of NICU transfer (minute)	39	21 (17–25)	46	24 (20–34)	15	28 (24–34)
No manipulation (seconds)	38	44 (8–104)	13	11 (2–47)	6	3 (1–17)
- % of analyzed time		4 (1–13)		1 (0–4)		0 (0–1)
Duration of single SI (seconds)	16	7.4 (6.1-8.2)	27	8.2 (5.8-9.4)	13	8.1 (5.8-9.9)
Duration of single intubation attempt (seconds)		46		52 (27–64)		45 (24–60)

For most of the time some interventions were performed on the infants. On 57 of the 111 infants there was a short time (25 s (6–79) in median) without any manipulation. As shown in Table 1, the more immature the infants were, the less the likelihood of manipulation.

### Prevention of heat loss

All but 3 infants were initially placed under the radiant warmer in a towel and were dried; the remaining 3 were placed in a plastic wrap without drying. Fourteen of the dried infants were also placed in a plastic wrap (1 in LGA, 5 in VLGA and 11 in ELGA). Towels were changed on 41 infants twice and on 2 infants three times.

Temperature was measured on 54 (16 in LGA, 25 in VLGA, 13 in ELGA) infants once (median 1–1 times) after a median of 13 (5–21) minutes.

### Assessment of infant’s condition

**Fig. 1:**
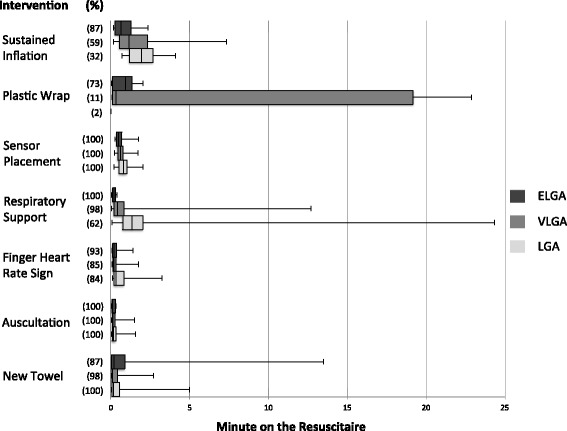
Time to first use of intervention I. Shown are how often the first 7 interventions were given (n in parenthesis - % of all of the groups). For each intervention the time point of first occurrence is given as median (horizontal line in the middle of each box), 25th and 75th percentiles (top and bottom of each box) and minimum/maximum (whiskers mark) of all analyzed recordings

Auscultation was performed on all newborns for a median of 3.9 (2–6) minutes, which represents between 0.4 and 70.1 % of the analyzed time. Pulse frequency was visualized on 95 infants (42 in LGA, 39 in VLGA and 14 in ELGA) and for 18.4 % (8.5–33.7) of the total auscultation time. A pulse-oximetry sensor was placed on all infants after a median of 37 (28–52) seconds (Fig. 1).

### Respiratory support

A total of 91 (83 %) of infants received respiratory support, starting in a median of 0.5 min after arrival under radiant heater (Fig. 1) and for a median duration of 22 min (Table 2). The more immature the infant, the earlier the respiratory support was started (Fig. 1). No newborn required chest compressions.

**Table 2:** Detailed respiratory support durations

	LGA		VLGA		ELGA	
	n	Total Time^a^	n	Total Time^a^	n	Total Time^a^
Total time spent for respiratory support	31	17.9 (11.6-23.2)^b^	45	22.8 (16.6-30.5)	15	28.4 (24–31.5)^c^
CPAP						
-CPAP through face mask or endotracheal tube^d^	31	9.6 (6.8-14.7)	45	10.9 (7.4-19.1)	15	9.7 (5.2-19.4)
-CPAP through nasopharyngeal tube	24	7.8 (4.1-12.2)	35	7.8 (4.3-13.7)	7	13.6 (3–15.8)
Mechanical Ventilation						
-Consecutive inflations	9	0.5 (0.2-1.5)	18	1.1 (0.5-2.8)	12	1.5 (0.6-2.7)
-Through endotracheal tube	1	11.6	7	12.1 (5.7-20.1)	9	14.9 (7.7-24.6)

^a^duration is presented in minutes [median (IQR)]

^b^p between LGA and VLGA < 0.05

^c^p between LGA and ELGA < 0.001

^d^Non-mechanical ventilation delivered in newborns intubated for surfactant application purposes only (intubation – surfactant application – extubation)

A sustained inflation was performed on 56 newborns 3 times (2–3) in median. Duration of single sustained inflation was in median 8 s (5.9–9.4) (Table 1).

Mechanical ventilation (through an endotracheal tube or as consecutive inflations via a face mask) was performed on 41 infants within a median of 2.4 min after arrival at the resuscitaire.

**Fig. 2:**
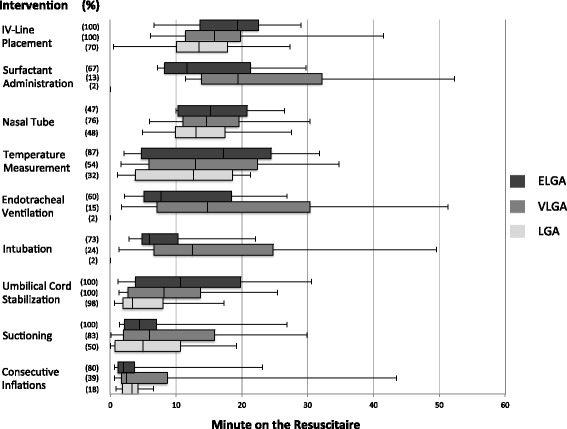
Time to first use of intervention II. Shown are how often the rest of the interventions were given (n in parenthesis - % of all of the groups). For each intervention the time point of first occurrence is given as median (horizontal line in the middle of each box), 25th and 75th percentiles (top and bottom of each box) and minimum/maximum (whiskers mark) of all analyzed recordings

**Fig. 3:**
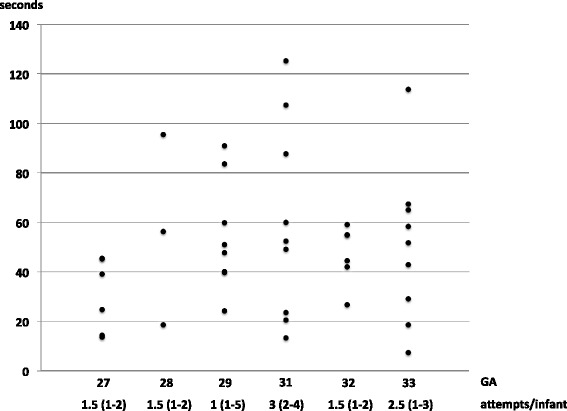
Time taken to intubate according to GA. Shown are the intubation attempts according to gestational age (GA). Every dot represents the duration of an intubation attempt (including successful and unsuccessful attempts)

Less than a quarter of the infants (1 from LGA, 11 from VLGA and 11 from ELGA) were intubated in the delivery room. The first intubation attempt was performed in a median after 9.3 min (Fig. 2). For successful intubation a median of 2 attempts (range 1–2) were required with a median duration of 47 (25–60) seconds (Fig. 3).

A total of 17 infants received exogenous surfactant (1 from LGA, 6 from VLGA and 10 from ELGA) after a median of 15 min (Fig. 2). Only 5 (29 %) of them were extubated after surfactant administration (INSURE). Duration of surfactant administration was 392 s in LGA, 76 (35–115) in VLGA and 69 (44–113) in ELGA.

A facemask was replaced by a naso-pharyngeal tube on 66 infants after 14 (10–18) minutes to administer CPAP for subsequent transport to the NICU (Fig. 2). Time spent for nasopharyngeal tube placement was between 6 and 65 s per attempt.

78 infants (71 %) were suctioned a median of 2 (1–4) times, lasting 7 (5–13) seconds per attempt.

### IV-line placement

An attempt to place an IV-line was performed on 100 infants (39 on LGA, 46 on VLGA and 15 on ELGA) (Table 3). On 4 LGA infants after three attempts in median (difference range: 2–5), an IV-line could not be placed and these infants were transported without an IV-line to the NICU. No infant received an umbilical cord catheterization in the DR.

**Table 3:** IV-Line placement data

	LGA	VLGA	ELGA
Infants who received an IV-line (%)	35 (70)	46 (100)	15 (100)
Attempts /infant	1 (1–5)	1 (1–7)	1 (1–3)
% of success from first attempt	64	59	60
Total time (minutes)	4 (3–6)	5 (3–7)	5 (3–6)
Timepoint at completion of the IV-Line^a^	13 (10–18)	15 (11–19)	19 (13–22)
Time spent only for successful attempt (minutes)	3 (2–4)	3 (3–4)	3 (3–5)

^a^timepoint is presented in minute on the resuscitaire [median (IQR)]

## Discussion

The more immature the infant the more likely some form of medical support during postnatal transition will be required. The quality of DR-management does have a great impact on the outcome of these vulnerable infants. Whereas some interventions have been investigated in clinical studies, little is known about the procedure followed during the entire postnatal management. According to our knowledge, the present study is the first detailed analysis of DR-interventions on preterm infants.

Using video analysis as a tool to study process quality, we show that interventions differ not only between but also within similar age groups. By detecting deviations from local guidelines we have provided a tool for improving the quality of DR-management and we have defined areas so as to achieve a quality improvement with goals that can be quantified and thus its achievement verified.

### Variation with respect to gestational age

Immaturity often results in disturbances of respiratory adaptation and subsequent need of medical support. In the present study, most of the infants received respiratory support, however, time of initiation varied with gestational age. Whereas respiratory support started immediately after arrival at the resuscitaire on ELGA-infants, it started significantly later on VLGA. These findings raise an interesting question; should respiratory support be considered a prevention (with an immediate initiation without any symptoms) or a treatment (initiated if first symptoms are present) of respiratory distress? Prematurity and delivery by c-section are two major risk factors for subsequent development of respiratory distress. Early CPAP is more effective in preventing than in treating respiratory distress [25]. Since almost all VLGA infants required some respiratory support at some point in time, it could be argued, that initiation of CPAP should start as soon as possible on infants with a gestational age below 33 weeks. That aspect could be considered in future guidelines, however appropriate trials are needed.

A similar variation between different GA-groups was found for sustained inflations. Whereas the majority (87 %) of ELGA infants received a SI, only 59 % of VLGA and 32 % of LGA received a SI. Furthermore, SI was administered within the first minute on ELGA and VLGA, but only after 7 min on LGA. During a SI the airway pressure is increased, pressing the lung fluid into the interstitial space. Studies on rabbits showed benefit when SI is administrated prior to the onset of breathing, e.g. in fluid filled lungs [26]. The observed practice of applying a SI after 7 min is questionable since there is no data concerning a benefit but rather possible side effects of administering this airway pressure for a long time in (partially) air-filled lungs. According to the present analysis, only infants with a gestational age below 31 weeks were considered to breathe insufficiently by the caregivers and thus received SI. However, studies have shown that breathing is present in smaller infants and the need for SI could be questioned.

### Adherence to guidelines

SI – If administered appropriately – seems to be effective in preventing intubation in DR [9]. Thus, SI’s were incorporated into our local neonatal resuscitation guidelines in 2010. However, the present analysis shows that clinical practice differs from the current recommendation. Time of initiation and duration of SI varied in and between different GA-groups. On some ELGA infants a single inflation lasted up to 19 s and some infants received up to 15 SI. To date, there is a lack of studies on SI and little is known regarding the optimal duration, time point or pressure [9, 27]. Since the present study was not designed to analyze the outcome of the DR management it does not provide any data regarding the incidence of pneumothorax. In the light of limited evidence on the benefits or side effects it seems to be important to adhere strictly to local recommendations, which are based on existing clinical data.

Local guidelines recommend, when surfactant administration is considered necessary, that infants should be intubated after a period of stabilization, surfactant should be given as a slow bolus during spontaneous breathing and infants should be extubated on CPAP [modified less invasive surfactant administration (LISA)-procedure] [28]. Our analysis showed that the management we followed deviates from these guidelines. About a quarter of infants that were intubated did not receive surfactant even though they met criteria for surfactant administration. Furthermore, only about 29 % of infants were extubated after surfactant administration. To improve quality of DR-management it will be necessary to detect reasons for not complying with the current guidelines [6]. As shown by Schilleman et al., guidelines could be too complicated [29]. According to our analysis, the duration of surfactant administration also varied significantly. Whereas slow surfactant infusion has been shown to be inefficient on ventilated infants, there is no sufficient data on the optimal duration of surfactant administration during spontaneous ventilation. For LISA-procedure with a feeding tube a time of 1–3 min has been suggested [28, 30]. However, it remains unclear whether it will be appropriate for intubated infants as well.

Monitoring of vital parameters represents an important aspect of DR-management [16]. Since measurement of oxygen saturation is a prerequisite to guide oxygen therapy, the request for an immediate placement of saturation sensor after arrival of the infant was added to our local guidelines a few years ago. Our analysis showed that the sensor was placed in a median after 37 s – thus we achieved sufficient adherence to our guidelines with regard to saturation measurement. Our guidelines further recommend that during auscultation the actual heartbeat must be shown by a finger movement until a sufficient sensor signal is available. That happened in approximately 86 % of cases. Since heart rate is an important parameter to evaluate efficacy of postnatal adaptation, an improvement is needed.

### Defining fields for improvement

International guidelines suggest 20 s as an appropriate time for intubation; however, this recommendation is based on limited data. Our current analysis showed great variation regarding duration of intubation, lasting up to 2 min. In approximately 11 % of all intubations attempts, time was as recommended in current guidelines. Interestingly, time needed for intubation did not differ significantly between ELGA and VLGA. It could be argued that the lack of medication for intubation and surfactant administration is a reason for the longer time needed for intubation. On the other hand, our data supports ongoing discussions regarding the optimal duration for intubation [31–33]. Further research is needed to find a correlation between a critical time and outcome. Even if the current analysis was not designed to study side effects of intubation, no major complications were observed. Nevertheless, improving the intubation skills of the caregiver has been defined as an enhancement target in our institution. The aim being to reduce median intubation time down to 30 s.

Stress of the newborn is associated with increased energy demand and oxygen consumption, which will lead to severe complications on preterm infants [34, 35]. Therefore, it is the general consensus to minimize stress and to apply the principles of optimal handling on these vulnerable infants. Our analysis showed that the first minutes of extra-uterine life are rather stressful for preterm; almost all infants experienced some kind of handling during some point of postnatal adaptation. Considering the importance of undisturbed adaptation we decided to re-evaluate the necessity of all handling procedures during DR-management. In future studies we aim to investigate, whether prolonged periods of no handling will have a beneficial effect on postnatal adaptation.

An important aspect to reduce energy consumption is appropriate temperature management. Our analysis shows good adherence to our internal guidelines, which recommends plastic wrap on infants with a birth weight below 1500 g. Whereas low admission temperatures are easily prevented by plastic wrap, there is a danger of overheating. Thus, according to our guidelines it is recommended to check the temperature. In the current analysis only about 49 % of the infant’s temperature was measured in a median after 13 min. Since we did not record the NICU admission temperature in the present study, we are not able to give any data concerning the effect.

We have recently described great variations concerning the time needed for IV-placement in term newborns [22]. The present study shows similar results for preterm infants. Interestingly, the number of attempts and the duration was lowest on the most immature infants. This finding can be explained by the fact that IV-lines were placed by more experienced caregivers on these infants, whereas lines were placed by more junior staff in more mature infants. Nevertheless, improving skills of placing IV-lines have to be improved in the future. Since placement of IV-lines represents a common procedure in neonatology, it would be of interest to compare different centers or even the policy of nurses versus medical doctors.

Whereas routine suctioning has been recommended in the past, it has been abandoned in both current international and local guidelines. Nevertheless, 70 % of the newborns were suctioned during the time period of current analysis. In a previous publication we have analyzed the effects of suctioning in term infants and did not find any beneficial effect (but also no side-effects) [24]. Thus, future training of our staff will focus on preventing routine suctioning.

## Conclusions

Avoidance of stress during postnatal transition seems to be important, especially in the preterm population. Thus, manipulation should be restricted to a required minimum; delivery room management should be focused on supporting transition rather than resuscitation. Whereas that approach seems to be a simple mission, daily practice is rather different. Video monitoring of delivery room management, combined with a subsequent structured analysis and feedback represents an important tool to discuss the “appropriateness” of all administered interventions.

The present study shows that analyzing of different processes of DR-management is feasible in great detail. Data shows variations in care of the infants which could have an impact on subsequent outcome. In the past, clinical studies were performed to examine the effect of a single intervention of DR-management. Despite the scientific advantage of this approach, it has a major limitation since it neglects the heterogeneity of DR-management in the daily routine. By analyzing all processes of current management, variations can be found which could be of clinical relevance. As a consequence habits can be changed. A subsequent re-evaluation of processes and outcome will show whether changes have improved outcomes. Even more interestingly, benchmarking of different centers can be initiated, based upon a valid process analysis.

## Additional files

Additional file 1: Table S1. Exact duration of various events for every LGA, VLGA and ELGA that was analyzed. (XLSX 45 kb) Additional file 2: Figures S1, S2. The time point of first occurrence of an intervention for every LGA, VLGA and ELGA that the specific intervention was given. (XLSX 78 kb) Additional file 3: Table S2. Detailed respiratory support administration duration of every LGA, VLGA and ELGA. (XLSX 43 kb) Additional file 4: Figure S3. Duration of every intubation attempt (successful and unsuccessful) according to the GA. (XLSX 30 kb) Additional file 5: Table S3. IV-Line placement data according to GA. (XLSX 48 kb)

## Abbreviations

CPAP: Continuous positive airway pressure
c-section: Caesarean section
ELGA: Extremely low gestational age
GA: Weeks of gestation
INSURE: Intubation-surfactant-extubation
IV-line: Intravenous line
LGA: Low gestational age
LISA: Less invasive surfactant administration
SI: Sustained inflation
VLGA: Very low gestational age
